# Synergistic neuroprotective effects of Danshensu and hydroxysafflor yellow A on cerebral ischemia-reperfusion injury in rats

**DOI:** 10.18632/oncotarget.23272

**Published:** 2017-12-15

**Authors:** Hang Xu, WenXing Liu, TianLong Liu, Ning Su, Chao Guo, XiaoNa Feng, Fang Dou, Yi Ding, Lei Shi, AiDong Wen

**Affiliations:** ^1^ Department of Pharmacy, Xijing Hospital, The Fourth Military Medical University, Xi’an 710032, China; ^2^ Department of Radiation Oncology, Xijing Hospital, The Fourth Military Medical University, Xi’an 710032, China; ^3^ Department of Pharmacy, Guangzhou General Hospital of Guangzhou Military Command, Guangzhou 510010, China

**Keywords:** Danshensu, hydroxysafflor yellow A, synergistic effect, neuroprotective effect, ischemic stroke

## Abstract

Ischemic stroke is a common cerebrovascular disease with substantial morbidity and mortality worldwide. However, therapeutic options to minimize the cerebral ischemia-reperfusion (I/R) injury are limited. In China, combination of herb Danshen (*Salvia miltiorrhiza Bge*) and Honghua (*Carthamus tinctorius L.*) is effective for stroke treatment in patients but its underlying mechanism requires further investigation. Our study was conducted to evaluate and explore the synergistic effects of two herb ingredients Danshensu and hydroxysafflor yellow A (HSYA) on cerebral ischemia-reperfusion (I/R) injury in rats. Rats were randomly assigned to the following five groups: sham group, model group, Danshensu group, HSYA group, and Danshensu+HSYA group. Under our experimental conditions *in vitro*, oxygen-glucose deprivation (OGD) model was established to determine the synergistic neuroprotective effects of Danshensu and HSYA. With such methods as neurological deficits scoring, TTC, HE and TUNEL staining, and ELISA detection, the results demonstrated that administration of either Danshensu or HSYA improved neurological defects and alleviated pro-inflammatory and oxidative stress reactions. Notably, combination of Danshensu and HSYA exerted more effective results than that used alone. Furthermore, western blot analysis results showed that Danshensu and HSYA combination displayed synergistic regulation on TLR4/NF-κB and Nrf2/HO-1 pathways. Consistently, Danshensu +HSYA group exhibits better neuroprotection in primary neurons with OGD model compared with Danshensu or HSYA group. Taken together, we found for the first time that Danshensu plus HSYA could achieve remarkable synergistic neuroprotective effects on I/R injury, which is related to the anti-inflammatory and antioxidant pathways.

## INTRODUCTION

Stroke is one of common lethal cerebrovascular diseases with substantial morbidity and mortality around the world [1]. Thrombolytic therapy remains the only beneficial therapy for stroke currently. In efforts to find drugs to effectively prevent and treat stroke, more and more attention has been focused on the chemicals extracted from traditional Chinese herbs [2]. In traditional Chinese medicine (TCM), a number of herbs are paired together in order to enhance the therapeutic effects. Synergy may occur when two or more herbal ingredients are combined, with more significant effects than administered alone [3, 4]. Danshen (*Salvia miltiorrhiza Bge*) and Honghua (*Carthamus tinctorius L.*) are valued Chinese traditional herbs for the treatment of cardiovascular diseases by multiple pharmacology effects such as anti-inflammatory and antioxidant role [5, 6]. In the clinical practice, Danshen and Honghua combination (such as DanHong injection, a Chinese material standardized clinical product) has been widely employed for cerebrovascular patients [5, 6].

Danshensu derived from *Salvia miltiorrhiza* is a main active ingredient in water-soluble components, and hydroxysafflor yellow A (HSYA) derived from the dried flower of *Carthamus tinctorius L.* is one of its bioactive components [7, 8]. Studies from our laboratories and others have reported that either Danshensu or HSYA can protect against I/R injury through anti-inflammatory and antioxidant pathways [7–9]. In addition, Danshensu or HSYA can readily permeate the blood brain barrier after oral administration of Danshen or Honghua extract to rats [10, 11]. Our previous investigation has revealed that Danshensu and HSYA in combination could exert better protective effects than used alone on myocardial ischemic-reperfusion injury in rats [12]. However, synergistic neuroprotective effects of Danshensu and HSYA upon cerebral I/R injury and their mechanisms are little known to date.

Inflammation and oxidative stress are the main pathophysiological processes concerning I/R injury and contribute to increased morbidity and mortality [13, 14]. Anti-inflammation can diminish the damage of ischemic brain tissue through removing the inflammatory factors and repairing the damaged tissue [15]. Toll-like receptor 4 (TLR4) from the pattern recognition receptor family is kind of transmembrane protein [16]. Its activation leads to nuclear factor kappa-light-chain-enhancer of activated B cells (NF-κB). Additionally, neuronal cells defense themselves against toxic reactive oxygen species (ROS) insult *via* anti-oxidation enzyme system which is mainly modulated by nuclear factor erythroid-2-related factor 2 (Nrf2) with Nrf2 related pathway a therapeutic target for stroke [4, 17]. Our previous studies also demonstrated that Nrf2/heme oxygenase-1 (HO-1) pathway is a potential target for the neuroprotection against stroke [18, 19]. Combined with the important role of Inflammation and oxidative stress in stroke, we hypothesized that the combined use of Danshensu and HSYA may significantly increase the therapeutic effect on stroke through TLR4/NF-κB and Nrf2/HO-1 pathways.

In our experiments, the protective function of Danshensu, HSYA and their combination on cerebral infarction were evaluated using rat model of stroke and cortical neurons of oxygen-glucose deprivation (OGD) for the purpose of elucidating the mechanism of the synergy protection of Danshensu and HSYA against ischemic stroke.

## RESULTS

### Danshensu and HSYA synergistically improved neurological deficit and cerebral infarction

**Figure 1:**
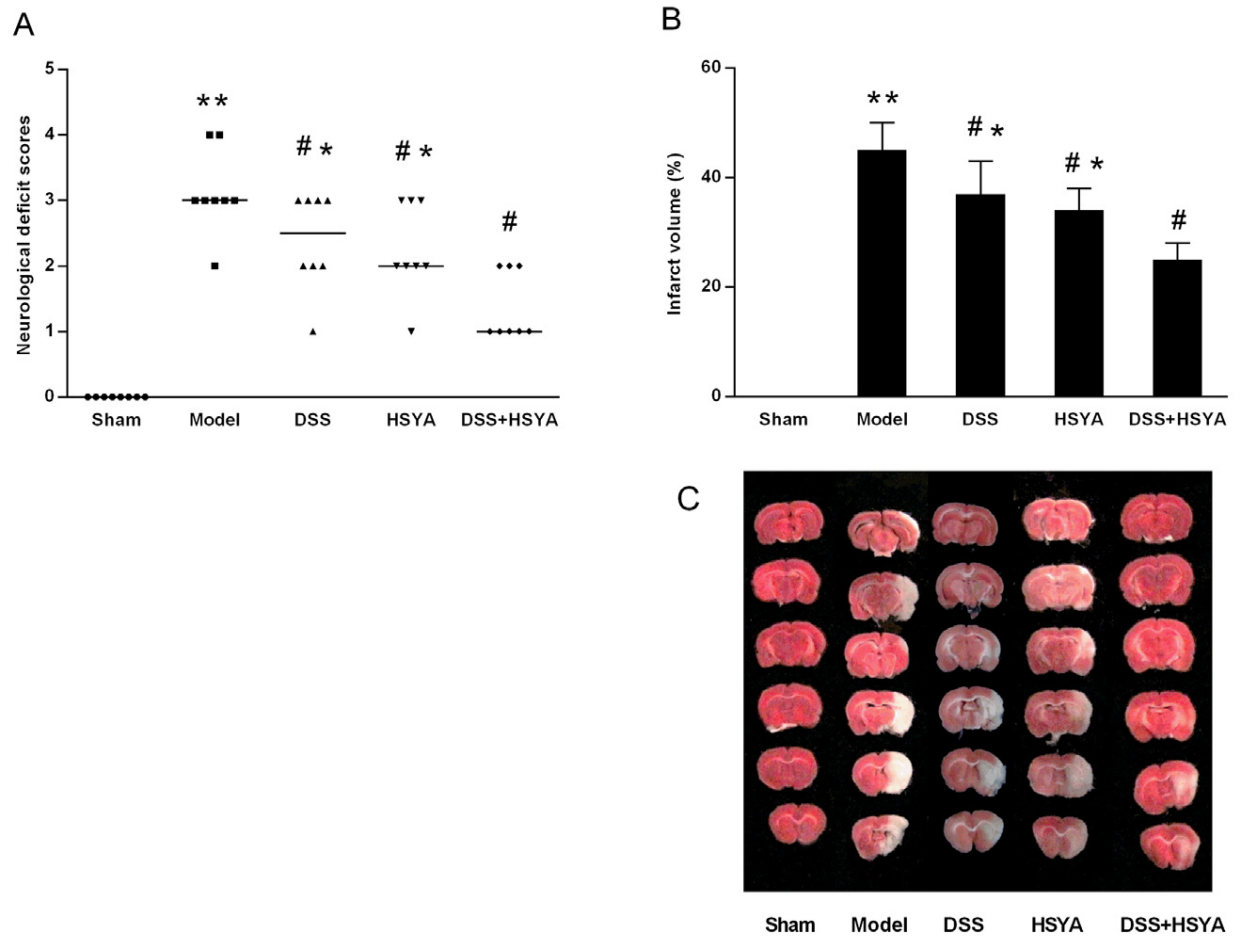
Neurological deficit evaluation **(A)** and infarct volume ratio by TTC staining **(B, C)**. (A) The neurological scores after MACO in each group (n=8). Neurological scores were expressed as median (range); ^*^P < 0.05, ^**^P < 0.01 compared with model group. (B, C) The infarct volume ratios in each group. ^#^P < 0.05 vs. model group; ^*^P < 0.05 vs. DSS+HSYA group. ^**^P < 0.01 vs. sham group.

Neurological scores were assessed at 48 h after reperfusion as shown in Figure 1A. The scores of Danshensu+HSYA group were remarkably higher than those in model group (P<0.05). While 2, 3, 5-triphenyltetrazolium chloride (TTC) staining indicated that the infarct volume in all treatment groups was significantly decreased compared with model group, Danshensu+HSYA group demonstrated the best protective effects (P<0.05). The representative coronal sections from different groups were exhibited in Figure 1B, 1C. Both the neurological deficit examination and infarct volume by TTC staining demonstrated that Danshensu and HSYA in combination synergistically protected neurological deficit and reduced infarction volume after stroke.

### Danshensu and HSYA in combination produced better protective effects on ischemic injury and neuronal apoptosis than each used alone

**Figure 2:**
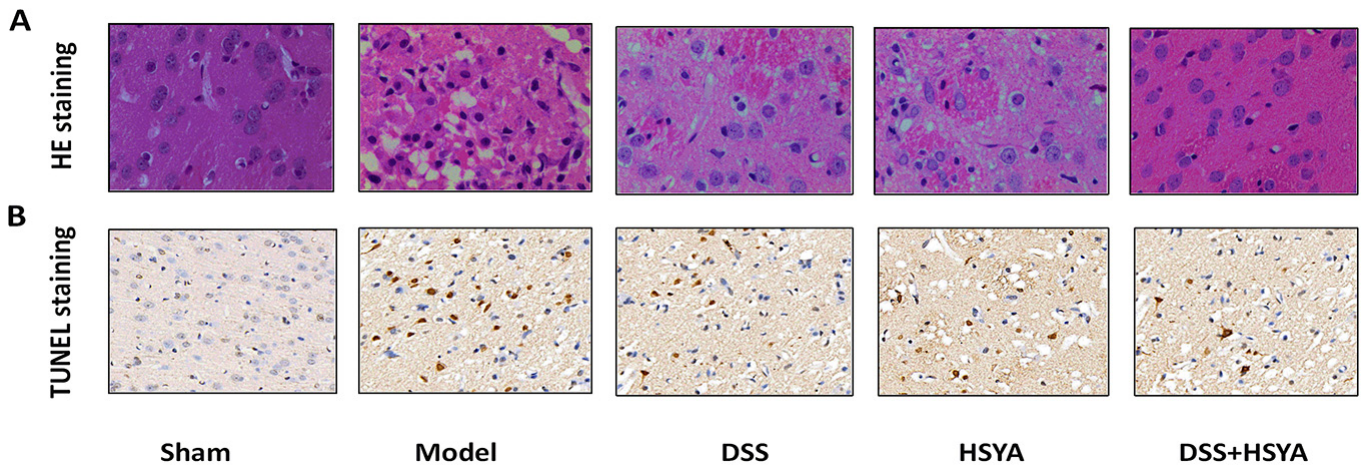
HE staining **(A)** and TUNEL assay **(B)**. (A) The morphological changes of cell swelling and pyknotic nuclei after 48 h of reperfusion in groups by HE staining. (B) TUNEL positive cells in cerebral cortex at 48 h of reperfusion in five groups. (400 magnifications).

Figure 2A shows the morphological features of neurons in each group by Hematoxylin and eosin (HE) staining. Histopathological abnormalities were not found in sham group and their neurons were arranged orderly with abundant cytoplasm and clear nucleolus. On the contrary, most neurons became shrunken and triangulated pyknotic nuclei were observed in the damaged area in model group after I/R. After the administration of Danshensu and HSYA the morphological changes took place in decreased cell swelling and pyknotic nuclei, with the most apparent reduction in Danshensu+HSYA group.

Apoptotic cells were detected by Terminal deoxynucleotidyl transferase-mediated dUTP-biotin nick end labeling (TUNEL) (Figure 2B). In model group, a significant increase of TUNEL-positive cells was observed in the cortex area. Moreover, TUNEL-positive cells were considerably diminished in treatment groups compared with those in model group. There were more TUNEL-positive cells in both Danshensu group and HSYA group than Danshensu+HSYA group.

### Danshensu and HSYA synergistically attenuated inflammation and oxidative stress

In our investigation, results from tumor necrosis factor (TNF-α), interleukin 1 beta (IL-1β) and interleukin 6 (IL-6) assessment demonstrated that of Danshensu or HSYA treatment has an anti-inflammatory effect for stroke. The levels of TNF-α, IL-1β and IL-6 were increased markedly in model group compared with sham group (P<0.05). Conversely, all of the pro-inflammatory cytokines were reduced in treatment groups with the best effects in Danshensu+HSYA group. Furthermore, there were significant decreases in the activities of superoxide dismutase (SOD) and glutathione peroxidase (GSH-Px) with a remarkable increase in malondialdehyde (MDA) content compared with sham group after middle cerebral artery (MCAO) modeling (Table 1). The case was improved noticeably with Danshensu or HSYA administration, whereas Danshensu+HSYA treatment enforced the strongest regulation on the activities of these oxidase and levels of oxidative product (P<0.05).

**Table 1:** Levels of inflammation and oxidative stress related factors at 48 h after MCAO in each group. Data are expressed as means ± SD (n = 8)

GROUPS	TNF-α	IL-1β	IL-6	Sod	GSH-Px	MDA
	(Pg/ml)	(Pg/ml)	(Pg/ml)	(U/mg)	(U/mg)	(μmol/mg)
Sham	57.6 ± 2.3	15.5 ± 1.2	65.6 ± 4.3	163.4 ± 9.5	73.1 ± 7.4	36.5± 8.7
Model	212.3 ± 13.8^**^	56.2 ± 3.1^**^	272.1 ± 10.8^**^	88.6 ± 8.4^**^	49.6 ± 4.7^**^	63.6± 13.5^**^
DSS	113.2 ± 12.0^#,*^	30.6 ± 3.4^#,*^	133.9 ± 11.1^#,*^	129.3 ± 9.7^#,*^	63.8 ± 7.5^#,*^	45.38± 8.7^#,*^
HSYA	114.7 ± 12.7^#,*^	27.6 ± 2.1^#,*^	124.7 ± 2.7^#,*^	121.9 ± 12.1^#,*^	57.4 ± 4.9^#,*^	53.02± 12.5^#,*^
DSS+HSYA	79.4 ± 6.1^#^	20.1 ± 3.3^#^	89.4 ± 6.1^#^	145.3 ± 5.5^#^	71.1±4.1^#^	41.60± 7.1^#^

^#^ P < 0.05 vs. model group; ^*^ P < 0.05 vs. DSS+ HSYA group. ^**^ P < 0.01 vs. Sham.

### Danshensu and HSYA suppressed TLR4 and NF-κB expression and enhanced Nrf2 and HO-1 expression

**Figure 3:**
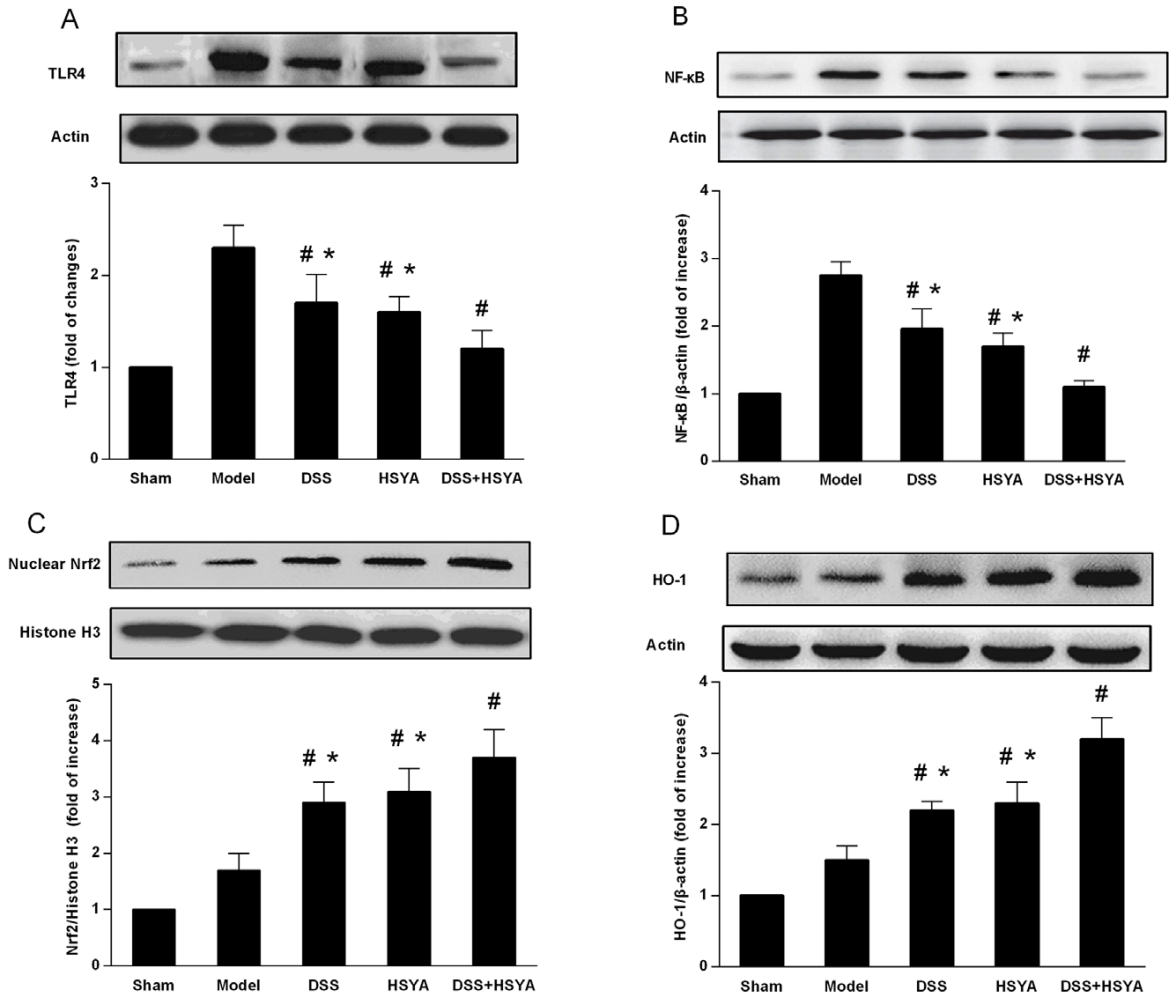
Western blot analysis of DSS and HSYA on the protein levels of **(A)** TLR4; **(B)** NF-κb; **(C)** Nrf2; **(D)** HO-1. ^#^P < 0.05 vs. model group; ^*^P < 0.05 vs. DSS+ HSYA group.

In order to further understand the anti-inflammatory and antioxidant mechanism of the combination effect of Danshensu and HSYA on the neuroprotection, we investigated the expressions of TLR4, NF-κB, Nrf2, and HO-1 in brain cortex tissues. The results from western blot analysis on brain cortex tissues demonstrated the curative functions of TLR4/NF-κB and Nrf2/HO-1 signaling pathways in the protection against stroke with Danshensu and HSYA used separately and in combination. As shown in Figure 3A and 3B, the protein levels of TLR4 and NF-κB in both Danshensu group and HSYA group were decreased, but those in Danshensu+HSYA group were dramatically reduced compared with ether Danshensu or HSYA group (P<0.05). Besides, administration with either Danshensu or HSYA moderately increased the expressions of Nrf2 and HO-1 but Danshensu plus HSYA achieved more significant increase (P<0.05) (Figure 3). Therefore, the most apparent synergistic effect of Danshensu and HSYA in TLR4/NF-κB and Nrf2/HO-1 pathways occurred through Danshensu plus HSYA treatment.

### Danshensu and HSYA synergistically protected neuronal cells against OGD-induced injury

**Figure 4:**
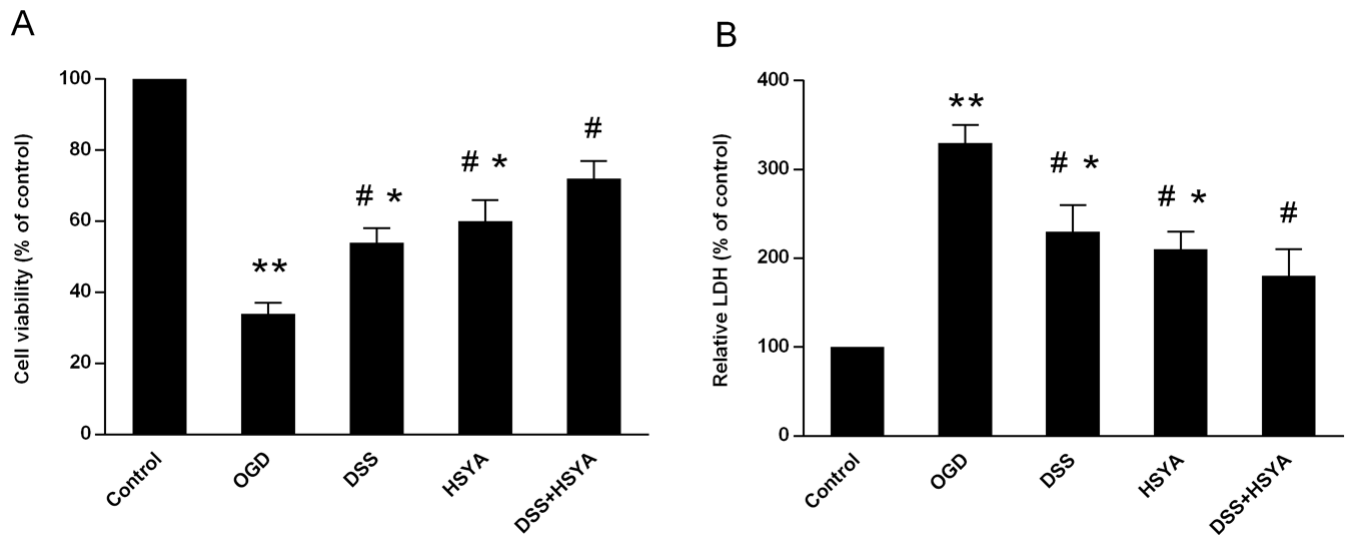
Cell viability **(A)** and LDH release **(B)** in primary culture of rat cortical neurons. The control group (normal cell group) was defined as 100%. Data represent means ± SD of triplicate independent experiments. ^#^P < 0.05 vs. OGD group; ^*^P < 0.05 vs. DSS+ HSYA group.

MTT assay was employed to evaluate the results of neuronal cells induced by OGD in the experiment. As shown in Figure 4, Danshensu and HSYA used separately or in combination defended neuronal cells under OGD condition but Danshensu plus HSYA treatment exhibited better curative effect on the viability increase compared with Danshensu or HSYA group (P<0.05) (Figure 4A). Moreover, the levels of lactate dehydrogenase (LDH) in the cell supernatant were increased in OGD group (P<0.05) but both Danshensu and HSYA groups exhibited lower LDH levels compared with OGD group (P<0.05). Their protective function was significantly enhanced in Danshensu+HSYA group in comparison with either Danshensu or HSYA group (P<0.05) (Figure 4B).

## DISCUSSION

Traditional Chinese medicinal herbs and their ingredients have been widely used as important therapeutic agents in China since ancient times. In clinical practice, they are commonly prescribed in combination to solve the complexity of a disease [3], such as the progression following ischemic stroke. In Traditional Chinese Medicine (TCM) theory, blood stasis syndrome is at the core of ischemic stroke [20, 21]. Danshen (*Salvia miltiorrhiza Bge*) and Honghua (*Carthamus tinctorius L.*) are popularly used to promote blood circulation to remove blood stasis, such as Buyang Huanwu decoction, a well-known TCM formula [22].

The present study demonstrated that Danshensu and HSYA as bioactive components of Danshen and Honghua possessed synergistic neuroprotection in MCAO and OGD model. These effects might be related to the anti-inflammatory and antioxidant pathways. The data of TTC staining, neurologic scores and HE staining showed that Danshensu and HSYA alone or in combination did decrease the I/R damage compared with model group but better cerebral protective effects appeared in Danshensu+HSYA group than Danshensu group or HSYA group. Accordingly, additive effects on cell viability and LDH were observed in primary neurons in OGD model with combined Danshensu and HSYA treatment. In our study, it is indicated that Danshensu and HSYA in combination exerted a synergic function on cerebral protection. Similarly, our previous study in rats and myocardial cell has also confirmed the synergistic effect of Danshensu plus HSYA in improving myocardial ischemia-reperfusion injury involved Nrf2/HO-1 pathwang [12].

Inflammation is one of the key pathogenetic factors in ischemic stroke [23]. In the process of stroke, I/R induced the production and secretion of apoptosis and such inflammation associated cytokines as TNF-α, IL-1β and IL-6 [24]. The increase of TNF-α, IL-1β and IL-6 in model rats was significantly alleviated through the treatments of Danshensu and HSYA alone or in combination. Notably, Danshensu plus HSYA effectively alleviated the increased contents of pro-inflammatory cytokines compared with Danshensu or HSYA treatment. Previous findings have demonstrated that the activation of innate immune receptors such as TLRs plays a decisive role in inducing inflammation [25]. Since NF-κB is a crucial modulator of inflammations during cerebral I/R injury [26], the organism of rats suffering from stroke could down-regulate inflammation and alleviate cerebral I/R injury by suppressing TLR4/NF-κB signaling pathway [27, 28]. Previous studies shown that HSYA has protective effects in I/R injury in animals by suppressing the TLR4-inducing pathway [29, 30]. Consistently, our comparative study of Danshensu and HSYA in the neuroprotection against stroke among single and combination groups suggest that Danshensu and HSYA in combination produced the most effective inhibitory effects on TLR4 and NF-κB expressions. Therefore, the synergistic neuroprotection of Danshensu and HSYA in combination may at least in part contribute to the inhibition of pro-inflammatory cascades during I/R.

Accumulating evidences have verified that oxidative stress is another kernel pathological factor in I/R injury and eliminating free radicals through SOD and GSH-Px could fight against oxidative stress damage induced by stroke [31]. The findings of our study in MCAO model showed that I/R increased lipid peroxidation and reduced the activities of such antioxidative enzymes as SOD and GSH-Px in brain. Danshensu+HSYA administration effectively heightened SOD and GSH-Px levels but diminished MDA content compared with Danshensu or HSYA treatment, indicating its synergistic effects on neuroprotection from the enhanced action of endogenous antioxidants. As Nrf2/HO-1 has an important role in endogenous antioxidative systems [32, 33], our investigation has confirmed that the activition of Nrf2/HO-1 can greatly exert neuroprotection against oxidative insults induced by I/R injury *in vitro* and *in vivo* [18, 19]. Other studies also demonstrated that Nrf2/HO-1 pathway is an important target for Danshensu or HSYA treatment for I/R injury [34, 35]. Similarly, our western blot analysis revealed that Danshensu+HSYA treatment group produced better effect in the increased levels of Nrf2 and HO-1 expression in comparison with Danshensu or HSYA alone treatment. This finding suggests that the enhanced therapeutic effect by Danshensu and HSYA combined administration may have a direct bearing on its better regulation on TLR4/NF-κB pathway.

In conclusion, Danshensu and HSYA individually has significant therapeutic effects on ischemic stroke but Danshensu and HSYA in combination appeared to possessed additive or synergistic protective effects, which may relate to the better regulation on TLR4/NF-κb and Nrf2/HO-1 pathways. We found for the first time that Danshensu+HSYA treatment resulted in the enhanced protection against cerebral ischemic insult by decreasing inflammation and improving antioxidant defense, suggesting Danshensu plus HSYA could effectively manifest synergistic neuroprotective effects on I/R injury through anti-inflammatory and antioxidant pathways. These findings provide the rational of applying Danshensu and HSYA in combination for stroke therapy. Further clinical investigation will be conducted into the development of pair herb regiments to effectively combat cerebrovascular complications.

## MATERIALS AND METHODS

### Animals

Male Sprague-Dawley rats (280±20g) were provided by the Experimental Animal Center of the Fourth Military Medical University. All experimental procedures were performed under the guidelines approved by the Ethics Committee for Animal Experimentation of the Fourth Military Medical University (Xi’an, Shaanxi, China). Food and tap water were available to the rats which were housed in air-conditioned animal quarters at 22.0±1.0°C and 40.0-50.0% humidity.

### MCAO model and grouping

Focal cerebral ischemia was induced through right MCAO with a poly-L-lysine-coated nylon suture [36]. The rats were anesthetized with 2.0 to 3.0% isoflurane and kept with 1.0% to 1.5% isoflurane under the condition of 70% N_2_O and 30% CO_2_. Then the rats were assigned to five groups according to the random number table: sham, model, Danshensu, HSYA, and Danshensu+HSYA group. In sham group, they were manipulated in the above mentioned way except for their middle cerebral artery occlusion. Two hours after the induction of ischemia, the filament in rats was slowly withdrawn.

Danshensu (99%, purity) and HSYA (98.0%, purity) were provided by Dong Ke Madison Bio-tech. Co., Ltd. (Xi’an, China). Rats in both sham and model groups were intraperitoneally injected with equal doses of 3 ml of 0.9% sodium chloride-diluted injection. Danshensu group and HSYA group were given 3 ml of 15 mg/kg Danshensu and 6 mg/kg HSYA diluted with 0.9% sodium chloride injection via intraperitoneal injection, respectively. Danshensu+HSYA group was injected 3 ml of 7.5 mg/kg Danshensu plus 3 mg/kg HSYA diluted with 0.9% sodium chloride injection intraperitoneally. The dose selection in each group was based on previous pharmacodynamics studies [37, 38]. The ratio between Danshensu and HSYA in the combined treatment was 2:1. This was derived from the clinical tradition such as Buyang Huanwu decoction and Danhong injection [6, 22], in which the ratio between Danshen and Honghua is from 2:1 to 3:1 when they are used together.

### Measurement of neurological scores and infarct volume

Neurological deficits of rats were evaluated blindly based on the Zea Longa standard [36] 48 h after the reperfusion with a 5-point scale system: 0 means no symptoms of neurological dysfunction; 1 means failure to fully stretch the contralateral torso and forelimb; 2 means movement to the ipsilateral side when held by the tail; 3 means action of falling over to the affected side; 4 means no spontaneous walking. For the measure of infarct volume, the removed brain in each group (n=8) was cut into coronal slices (2 mm) at 48 h after MCAO. The slices were incubated for 30 min in a 2% solution of TTC. The images of TTC-stained brain slices were then captured. Infarct volumes were expressed as percentages with the formula: ([total contralateral hemispheric volume]-[total ipsilateral hemispheric stained volume])/(total contralateral hemispheric volume)×100%.

### Histological examination and HE staining

Rats were euthanized and perfused with physiological saline solution at 4°C after 24 h of reperfusion. Then they were prepared with paraformaldehyde (4%, v/v) in phosphate buffered saline (0.1 M) buffer (pH 7.4). Brain block was embedded in paraffin and cut into 5 μm coronal sections after the brain was separated and fixed inparaformaldehyde (4%, w/v) for 24 h. HE staining was then conducted to observe the morphological changes of impaired neurons in the cerebral cortex (×400).

### TUNEL staining

The 5-μm-thick paraffin sections of tissues were prepared and the apoptotic myocardial cells were detected with a TUNEL assay kit (Roche, Germany) to assess DNA damage. The staining was executed based on the manufacturers’ directions. The positive cells exhibited green staining within the nucleus of apoptotic cells. The total number of TUNEL positive neurons in the ipsilateral hemisphere was counted in four different fields for each section. Images were captured with a fluorescence microscope (Olympus, Tokyo, Japan).

### Evaluation of pro-inflammatory cytokines

Animal blood in each group (n=8) was obtained from abdominal aorta aseptically at 48 h after reperfusion. The levels of TNF-α, IL-1β and IL-6 in serum were assessed using ELISA kits (Jiancheng Biological Engineering Institute, Nanjing, China). Protein concentration was measured by interpolation onto absorbance curves generated by recombinant TNF-α, IL-1β or IL-6 protein standards with an ELISA reader (Model ELX800, BioTek, USA).

### Assessment of antioxidant enzyme activity and MDA content

Rats in five groups (n=8) were sacrificed at 48 h after MCAO and their right cerebral cortex tissues were homogenized in 2 ml phosphate buffer (10 mM). As the activities of SOD and GSH-Px as well as the contents of MDA are essential indexes of evaluating antioxidative effects, they were quantified using test kit (Jiancheng Biological Engineering Institute, Nanjing, China). Then the optical density of samples was measured using microplate reader (Thermo Scientific, MA, USA).

### Western blot analysis

At 48 h after reperfusion, brain cortex tissues including the infarct area (n=4) were homogenized in each group. Protein concentration was measured by bicinchoninic acid method with bovine serumalbumin as the standard. Protein samples (60 μg) were loaded onto polyacrylamide gel, electrophoresed and transferred to a PVDF membrane. Then the membrane was incubated with primary antibody at a dilution of 1:1000 overnight after it was blocked with 5% BSAand incubated with the peroxidase-conjugated secondary antibody. The blots were obserbed with ECL-Plus reagent (Santa Cruz, USA).

### Primary culture of rat cortical neurons

Primary astrocyte cultures were obtained from neonatal rats as described previously [39]. Approximately 30,000 cells in 50 ml neurobasalmedium containing glutamine (1 mmol/L), 1% penicillin, streptomycin (Pen/Strep), and 10% fetal bovine serum were seeded into 6-well plates. After 2 h, 0.5 mlneurobasal medium containing the serum-free B27 supplement (2%), Pen/Strep, andglutamine were added to each well. After 2 days *in vitro*, 5 mM cytosinearabinofuranoside was added to inhibit glial proliferation. At 5 days *in vitro*, themedium was changed to fresh neurobasal medium containing B27. Neurons werecultured at 37°C in a humidified 5% CO_2_ atmosphere and used after 7 days *in vitro*.

### OGD model

To test the neuroprotective function of Danshensu and HSYA against ischemic injury, OGD model was employed as formerly described [18]. Cortical neurons from the brains of one-day-old rats were prepared. Around 30,000 cells from 50 ml neurobasal medium were seeded into 6 well plates and 0.5 ml neurobasal medium was added to each well 2 h later. After 48 h, cytosine arabinofuranoside (5 μM) was added to prevent from glial proliferation. To simulate ischemia-like atmosphere, cells were subjected to OGD for 60 min and incubated in the incubator with 95 % air and 5 % CO_2_ with different treatments for 24 h. The cells were divided into 5 treatments: control group (no OGD), OGD group, 80 μM Danshensu group, 80 μM HSYA group, and 40 μM Danshensu+40 μM HSYA group, and the dose was ascertained based on our previous experimental work [12]. OGD-induced cell death was quantified using the 3-[4,5-dimethylthiazol-2-yl]-2,5-diphenyltetrazolium bromide (MTT) assay as descibed previously [40].

### Statistical analysis

The statistical analyses were carried out with SPSS 16.0 (SPSS Inc., Chicago, IL, U.S. A). Neurological deficit scores were presented as the median (range) and analyzed through a nonparametric method (Kruskal–Wallis test) followed by the Mann–Whitney U-test using Bonferroni correction. All the other data were shown as mean ± standard deviation (SD). The differences among groups were made a comparison through one-way analysis of variance and Fisher’s post hoc test. A probability of P<0.05 was considered to be statistically significant.

## Abbreviations

GSH-Px: glutathione peroxidase
IL-1β: interleukin-1β
IL-6: interleukin-6
I/R: ischemia-reperfusion
HE: hematoxylin and eosin
HO-1: heme oxygenase-1
HSYA: hydroxysafflor yellow A
LDH: lactate dehydrogenase
MCAO: middle cerebral artery occlusion
MDA: malondialdehyde
MTT: 3-[4,5-dimethylthiazol-2-yl]-2,5-diphenyltetrazolium bromide
NF-κB: nuclear factor kappa-light-chain-enhancer of activated B cells
Nrf2: nuclear factor erythroid-2-related factor 2
OGD: oxygen and glucose deprivation
ROS: reactive oxygen species
SD: standard deviation
SOD: superoxide dismutase
TLR4: Toll-like receptor 4
TNF-α: tumor necrosis factor-α
TTC: 2,3,5-triphenyltetrazolium chloride
TUNEL: terminal deoxynucleotidyl transferasedUTP nick end labeling

## References

[1] Feigin VL, Roth GA, Naghavi M, Parmar P, Krishnamurthi R, Chugh S, Mensah GA, Norrving B, Shiue I, Ng M (2016). Global burden of stroke and risk factors in 188 countries, during 1990–2013: a systematic analysis for the global burden of disease study 2013. Lancet Neurol.

[2] Wu PF, Zhang Z, Wang F, Chen JG (2010). Natural compounds from traditional medicinal herbs in the treatment of cerebral ischemia/reperfusion injury. Acta Pharmacol Sinica.

[3] Ma X, Zheng C, Han L, Xie B, Jia J, Cao Z, Li Y, Chen Y (2009). Synergistic therapeutic actions of herbal ingredients and their mechanisms from molecular interaction and network perspectives. Drug Discov Today.

[4] Zhang Q, Wang J, Zhang C, Liao S, Li P, Xu D, Lv Y, Yang M, Kong L (2016). The components of Huang-Lian-Jie-Du-Decoction act synergistically to exert protective effects in a rat ischemic stroke model. Oncotarget.

[5] Wang H, Ren S, Liu C, Zhang X (2016). An overview of systematic reviews of danhong injection for ischemic stroke. Evid Based Complement Alternat Med.

[6] Guan Y, Yin Y, Zhu YR, Guo C, Wei G, Duan JL, Wang YH, Zhou D, Quan W, Weng Y, Xi MM, Wen AD (2013). Dissection of mechanisms of a chinese medicinal formula: danhong injection therapy for myocardial ischemia/reperfusion injury in vivo and in vitro. Evid Based Complement Alternat Med.

[7] Wei G, Yin Y, Duan J, Guo C, Zhu Y, Wang Y, Xi M, Wen A (2017). Hydroxysafflor yellow A promotes neovascularization and cardiac function recovery through HO-1/VEGF-A/SDF-1α cascade. Biomed Pharmacother.

[8] Yang Y, Xue X, Liu Z (2009). Protective effect of danshensu on focal cerebral ischemia-reperfusion injury in rats. Food Drug.

[9] Cao J, Chen Z, Zhu Y, Li Y, Guo C, Gao K, Chen L, Shi X, Zhang X, Yang Z (2014). Huangqi− Honghua combination and its main components ameliorate cerebral infarction with Qi deficiency and blood stasis syndrome by antioxidant action in rats. J Ethnopharmacol.

[10] Li Y, Zhang ZY, Zhang JL (2007). Determination of hydroxysafflor yellow A in rat plasma and tissues by high-performance liquid chromatography after oral administration of safflower extract or safflor yellow. Biomed Chromatogr.

[11] Zhang YJ, Wu L, Zhang QL, Li J, Yin FX, Yuan Y (2011). Pharmacokinetics of phenolic compounds of Danshen extract in rat blood and brain by microdialysis sampling. J Ethnopharmacol.

[12] Hu T, Wei G, Xi M, Yan J, Wu X, Wang Y, Zhu Y, Wang C, Wen A (2016). Synergistic cardioprotective effects of Danshensu and hydroxysafflor yellow A against myocardial ischemia-reperfusion injury are mediated through the Akt/Nrf2/HO-1 pathway. International journal of molecular medicine.

[13] Chamorro Á, Dirnagl U, Urra X, Planas AM (2016). Neuroprotection in acute stroke: targeting excitotoxicity, oxidative and nitrosative stress, and inflammation. Lancet Neurol.

[14] Ding Y, Qiao Y, Wang M, Zhang H, Li L, Zhang Y, Ge J, Song Y, Li Y, Wen A (2016). Enhanced neuroprotection of acetyl-11-keto-beta-boswellic acid (AKBA)-loaded O-carboxymethyl chitosan nanoparticles through antioxidant and anti-inflammatory pathways. Mol Neurobiol.

[15] Chen B, Liao WQ, Xu N, Xu H, Wen JY, Yu CA, Liu XY, Li CL, Zhao SM, Campbell W (2009). Adiponectin protects against cerebral ischemia–reperfusion injury through anti-inflammatory action. Brain Res.

[16] Caso JR, Pradillo JM, Hurtado O, Lorenzo P, Moro MA, Lizasoain I (2007). Toll-like receptor 4 is involved in brain damage and inflammation after experimental stroke. Circulation.

[17] Farrell-Dillon K, Chapple S, Fraser P, Mann G (2017). Long-term contribution of Nrf2 to behavioral recovery following focal cerebral ischemia-reperfusion injury. Free Radic Biol Med.

[18] Ding Y, Chen M, Wang M, Wang M, Zhang T, Park J, Zhu Y, Guo C, Jia Y, Li Y (2014). Neuroprotection by acetyl-11-keto-β-boswellic acid, in ischemic brain injury involves the Nrf2/HO-1 defense pathway. Sci Rep.

[19] Ding Y, Chen M, Wang M, Li Y, Wen A (2015). Posttreatment with 11-keto-beta-boswellic acid ameliorates cerebral ischemia-reperfusion injury: Nrf2/HO-1 pathway as a potential mechanism. Mol Neurobiol.

[20] Wang M, Chen M, Ding Y, Zhu Z, Zhang Y, Wei P, Wang J, Qiao Y, Li L, Li Y (2015). Pretreatment with β-boswellic acid improves blood stasis induced endothelial dysfunction: role of eNOS activation. Sci Rep.

[21] Kim H (2005). Neuroprotective herbs for stroke therapy in traditional eastern medicine. Neurol Res.

[22] Hao CZ, Wu F, Shen J, Lu L, Fu DL, Liao WJ, Zheng GQ (2012). Clinical efficacy and safety of buyang huanwu decoction for acute ischemic stroke: a systematic review and meta-analysis of 19 randomized controlled trials. Evid Based Complement Alternat Med.

[23] Hagberg H, Mallard C, Ferriero DM, Vannucci SJ, Levison SW, Vexler ZS, Gressens P (2015). The role of inflammation in perinatal brain injury. Nat Rev Neurol.

[24] Tuttolomondo A, Pecoraro R, Pinto A (2014). Studies of selective TNF inhibitors in the treatment of brain injury from stroke and trauma: a review of the evidence to date. Drug Des Devel Ther.

[25] Gesuete R, Kohama SG, Stenzel-Poore MP (2014). Toll-like receptors and ischemic brain injury. J Neuropathol Exp Neurol.

[26] Yu C, Li P, Qi D, Wang L, Qu HL, Zhang YJ, Wang XK, Fan HY (2017). Osthole protects sepsis-induced acute kidney injury via down-regulating NF-κB signal pathway. Oncotarget.

[27] Lan L, Tao J, Chen A, Xie G, Huang J, Lin J, Peng J, Chen L (2013). Electroacupuncture exerts anti-inflammatory effects in cerebral ischemia-reperfusion injured rats via suppression of the TLR4/NF-κB pathway. Int J Mol Med.

[28] Pan N, Lu LY, Li M, Wang GH, Sun FY, Sun HS, Wen XJ, Cheng JD, Chen JW, Pang JY (2017). Xyloketal B alleviates cerebral infarction and neurologic deficits in a mouse stroke model by suppressing the ROS/TLR4/NF-κB inflammatory signaling pathway. Acta Pharmacologica Sinica.

[29] Lv Y, Qian Y, Fu L, Chen X, Zhong H, Wei X (2015). Hydroxysafflor yellow A exerts neuroprotective effects in cerebral ischemia reperfusion-injured mice by suppressing the innate immune TLR4-inducing pathway. Eur J Pharmacol.

[30] Han D, Wei J, Zhang R, Ma W, Shen C, Feng Y, Xia N, Xu D, Cai D, Li Y, Fang W (2016). Hydroxysafflor yellow A alleviates myocardial ischemia/reperfusion in hyperlipidemic animals through the suppression of TLR4 signaling. Sci Rep.

[31] Liu Y, Zhang L, Liang J (2015). Activation of the Nrf2 defense pathway contributes to neuroprotective effects of phloretin on oxidative stress injury after cerebral ischemia/reperfusion in rats. J Neurol Sci.

[32] Alfieri A, Srivastava S, Siow RC, Cash D, Modo M, Duchen MR, Fraser PA, Williams SC, Mann GE (2013). Sulforaphane preconditioning of the Nrf2/HO-1 defense pathway protects the cerebral vasculature against blood–brain barrier disruption and neurological deficits in stroke. Free Radic Biol Med.

[33] Ding Y, Zhang B, Zhou K, Chen M, Wang M, Jia Y, Song Y, Li Y, Wen A (2014). Dietary ellagic acid improves oxidant-induced endothelial dysfunction and atherosclerosis: role of Nrf2 activation. Int J Cardiol.

[34] Li H, Song F, Duan LR, Sheng JJ, Xie YH, Yang Q, Chen Y, Dong QQ, Zhang BL, Wang SW (2016). Paeonol and danshensu combination attenuates apoptosis in myocardial infarcted rats by inhibiting oxidative stress: roles of Nrf2/HO-1 and PI3K/Akt pathway. Sci Rep.

[35] Liu SX, Zhang Y, Wang YF, Li XC, Xiang MX, Bian C, Chen P (2012). Upregulation of heme oxygenase-1 expression by hydroxysafflor yellow A conferring protection from anoxia/reoxygenation-induced apoptosis in H9c2 cardiomyocytes. Int J Cardiol.

[36] Longa EZ, Weinstein PR, Carlson S, Cummins R (1989). Reversible middle cerebral artery occlusion without craniectomy in rats. stroke.

[37] Chong Y, Wang T, Wang W, Zhang L, Li C, Yu P, Wang H, Fu F (2012). Down-regulation of P-glycoprotein expression contributes to an increase in Danshensu accumulation in the cerebral ischemia/reperfusion brain. Mol Med Rep.

[38] Zhu HB, Zhang L, Wang ZH, Tian JW, Fu FH, Liu K, Li CL (2005). Therapeutic effects of hydroxysafflor yellow A on focal cerebral ischemic injury in rats and its primary mechanisms. J Asian Nat Prod Res.

[39] Shi TY, Feng SF, Xing JH, Wu YM, Li XQ, Zhang N, Tian Z, Liu SB, Zhao MG (2012). Neuroprotective effects of Salidroside and its analogue tyrosol galactoside against focal cerebral ischemia in vivo and H2O2-induced neurotoxicity in vitro. Neurotox Res.

[40] Wang M, Li YJ, Ding Y, Zhang HN, Sun T, Zhang K, Yang L, Guo YY, Liu SB, Zhao MG (2016). Silibinin prevents autophagic cell death upon oxidative stress in cortical neurons and cerebral ischemia-reperfusion injury. Mol Neurobiol.

